# Transcriptional signature of host shift in the seed beetle *Zabrotes subfasciatus*

**DOI:** 10.1590/1678-4685-GMB-2023-0148

**Published:** 2024-02-05

**Authors:** Pedro Augusto da Pos Rodrigues, Juliana Ramos Martins, Bianca Corrêa Capizzani, Lucas Takashi Araujo Hamasaki, Zilá Luz Paulino Simões, Isabel Ribeiro do Valle Teixeira, Angel Roberto Barchuk

**Affiliations:** 1 University of Georgia, Department of Entomology, Athens, GA, USA.; 2 Universidade Federal de Alfenas (UNIFAL-MG), Instituto de Ciências Biomédicas, Departamento de Biologia Celular e do Desenvolvimento, Alfenas, MG, Brazil.; 3 Universidade de São Paulo, Faculdade de Filosofia, Ciências e Letras de Ribeirão Preto, Departamento de Biologia, Ribeirão Preto, SP, Brazil.; 4 Instituto Federal Sul de Minas (IFSULDEMINAS), Campus Poços de Caldas, MG, Brazil.

**Keywords:** Transcriptional signature, histone methylation, vitellogenin, host shift, seed beetle

## Abstract

In phytophagous insects, adaptation to a new host is a dynamic process, in which early and later steps may be underpinned by different features of the insect genome. Here, we tested the hypothesis that early steps of this process are underpinned by a shift in gene expression patterns. We set up a short-term artificial selection experiment (10 generations) for the use of an alternative host (*Cicer arietinum*) on populations of the bean beetle *Zabrotes subfasciatus*. Using Illumina sequencing on young adult females, we show the selected populations differ in the expression of genes associated to stimuli, signalling, and developmental processes. Particularly, the “*C. arietinum*” population shows upregulation of histone methylation genes, which may constitute a strategy for fine-tuning the insect global gene expression network. Using qPCR on body regions, we demonstrated that the “*Phaseolus vulgaris*” population upregulates the genes *polygalacturonase* and *egalitarian* and that the expression of an odorant receptor transcript variant changes over generations*.* Moreover, in this population we detected the existence of vitellogenin (Vg) variants in both males and females, possibly harbouring canonical reproductive function in females and extracellular unknown functions in males. This study provides the basis for future genomic investigations seeking to shed light on the nature of the proximate mechanisms involved in promoting differential gene expression associated to insect development and adaptation to new hosts.

## Introduction

The life cycle of most insects is closely associated with plants. The coleopteran clade Phytophaga (Superfamilies Chrysomeloidea and Curculionoidea), represented by approximately 135,000 species (Lawrence, 1982; Farrell, 1998), is among the richest in phytophagous insects. Together with Lepidoptera and Hemiptera, they represent the majority of herbivorous insects. These insects may use plants as food resource, mating site, oviposition and development site, and habitat during all or part of their life cycles. The use of a specific plant species as a host is first influenced by long distance-acting factors, such as chemical or visual cues. Thereafter, females usually evaluate the host and their choices are influenced by volatile, gustatory or tactile cues (Knolhoff and Heckel, 2014). Females are finally influenced by the chemical constituents of plants, among which are the plant secondary compounds, and those that may eventually be used to fuel reproduction (Ehrlich and Raven, 1964).

Plant use by insects is a phenotypically complex trait that is likely to be polygenic (Simon et al., 2015), where each step of a plant-insect interaction likely relies on a particular molecular framework. Studies carried out primarily with hemipterans and lepidopterans suggest that the phenotypic changes associated with host choice are made possible by variations in the expression levels of genes that code for chemosensory and detoxification systems (Simon *et al.*, 2015). In phytophagous Coleoptera, the acquisition of genes encoding enzymes capable of degrading compounds from plant cell walls has been found to be key to explain their success in consuming a variety of plant tissues including seeds, leaves, trunks, and wood as a food source (Farrell, 1998; McKenna et al., 2016, 2019). However, the identification of evolutionary patterns of herbivory in these insects, including the eventual occurrence of orthology, convergence or parallel evolution, is still limited by the paucity of studies in this area (Birnbaum and Abbot, 2020). Even less is known about the eventual adaptive role of genes or gene groups in the choice of a particular host, which could be evaluated by means of functional assays. Additionally, adaptation to a new host may result in the evolution of reproductive isolating barriers between populations thus eventually leading to speciation (Forbes et al., 2017).

Most phytophagous species in the subfamily Bruchinae of the family Chrysomelidae are hosted by plants in the Fabaceae family (Leguminosae) (Southgate, 1979; Johnson, 1981). Legume grains such as beans represent the main protein source for 75 % of the population in developing countries, and Bruchinae beetles are deemed responsible for 20 % of the loss in value of these grains (Cardona, 2004). Many species in this group can be reared with relative ease in captivity and are often used in artificial selection experiments. As a consequence, Bruchinae have become the focus of research on the genetic basis of niche occupation (i.e. adaptation to new environments) and host selection (e.g. Messina et al., 2009; Rêgo et al., 2019). In this case, host selection experiments can be conducted under conditions very similar to those encountered by these insects, i.e. stored seeds. In one of these studies, researchers found that strains of the cowpea seed beetle *Callosobruchus maculatus* selected to grow in lentils instead of its more commonly used host plant, the mung bean (*Vigna radiata*), differentially express genes related to detoxification, such as genes encoding beta-glucosidase and cytochrome P450 (Rêgo *et al.*, 2019, 2020). Interestingly, *C. maculatus* need of low expression levels of the beta-glucosidase gene for the use of *Vicia faba* was reported in the nineties after a comprehensive genetic and biochemical analysis of selected strains (Desroches et al., 1997). Detoxification genes were also found to be highly expressed in lepidopterans and hemipterans exposed to variations in secondary host compounds (Simon et al., 2015). Rêgo *et al.* (2019, 2020) notes that different groups of genes are found to be associated with host change, depending on the approach used, i.e. genomic sequencing, analysis of QTLs/SNPs or gene expression. In other words, different experimental approaches may yield non-overlapping results, and likely an incomplete idea of ​​the genetic basis of adaptation. These results illustrate the need for studies using similar approaches and a greater range of insect-plant systems, to allow the detection of generalizations and specificities regarding the interaction of environment-genotype-phenotype (fitness) in these systems, and in organisms in general (Oppenheim et al., 2015; Simon *et al.*, 2015; Birnbaum and Abbot, 2020).

*Zabrotes subfasciatus* Boheman (1833) (Chrysomelidae; Bruchinae; Schoch et al., 2020; https://eol.org/pages/1174683) is a beetle native to Central/South America, from where it has spread throughout the world, fundamentally to tropical regions, following approximately the regions that cultivate Fabaceae (Lucas, 1858; Valencia et al., 1986). As in other bruchid species, females lay eggs on the surface of a host seed. The larva that hatches after embryonic development pierces the tegument and develops inside the seed, feeding on the cotyledons and embryonic regions. After pupal development, adults emerge (after 26 days of development at 30-35 °C, Corrêa et al., 2021), look for host seeds, mate, and the cycle resumes, without the need to ingest food or water, which appears to be facultative (Howe and Currie, 1964; Southgate, 1979; Kingsolver, 2004; Corrêa *et al.*, 2020, 2021).

Research on the biology of host use by *Z. subfasciatus* shows that this species can grow on several Fabaceae species, but prefers bean (*Phaseolus vulgaris*) varieties, where it shows high fitness ​​(Meik and Dobie, 1986; Teixeira and Zucoloto, 2003, Teixeira *et al.*, 2008, Corrêa et al., 2020). In *Cicer arietinum* (chickpea), for example, *Z. subfasciatus* has the worst fitness values ​​(Teixeira and Zucoloto, 2003), and the seeds of this host are the least chosen in behavioral tests (unpublished material from our laboratory). After 7 generations of selection, however, performance in this seed increases (Teixeira *et al.*, 2008). In other species of the Fabaceae family, such as *Vicia faba, Z. subfasciatus* does not even lay eggs (Corrêa *et al.*, 2020). Results obtained by our research group suggest that the choice of hosts by this beetle is defined by the females and requires the participation of volatiles emanating from the seeds (unpublished material from our laboratory).

The difficulty encountered by populations of *Z. subfasciatus* to use chickpeas as a host may reside in that the seeds of this Fabaceae, despite having a protein content similar to those of their usual host, the carioca beans, are larger, heavier, and have a different texture and a significantly lower amount of water (Teixeira and Zucoloto, 2003; the emission of deterrent volatile organic compounds seems to also be responsible for the observed difficulty in acceptance (unpublished material from our laboratory). The eventual host shift, from beans to chickpeas, in addition to being time consuming, is accompanied by a significant fitness decrease (mainly the reproductive capacity) of the population in the new host. Once the new host (chickpea) is “conquered”, *Z. subfasciatus* populations show fitness comparable to those shown in the usual host, the common bean (Teixeira *et al.*, 2008), indicating adaptation.

The adaptation to the use of a new host is a dynamic and progressive process. Early and later steps of the process may be underpinned by the alteration of different (structural or functional) aspects of the insect genome (Langmüller and Schlötterer, 2020). Here, we tested the hypothesis that the early steps of adaptation for the use of a new host for oviposition and development are underpinned by a shift in its gene expression landscape, in particular for genes associated with finding new hosts, such as those that intermediate functioning of the chemosensory system and genes associated with consuming a new host, such as detoxifying genes. We set up a short-term artificial selection experiment for the use of an alternative, less preferred host, using populations of the bean beetle *Z. subfasciatus*. Our results show the selected populations have distinct transcriptomic landscapes, which includes significant differential expression of transcript variants, and pinpoint the dynamics of transcription patterns of a subset of genes, critical for the development of life-history traits of *Z. subfasciatus*.

## Material and Methods

### Beetle populations and artificial selection

A laboratory *Z. subfasciatus* stock population (~ 6,000 individuals), collected from bean seed stocks in 1997 in Ribeirão Preto, São Paulo, Brazil, was used for this study. The level of genetic differentiation in the Brazilian populations of this species is low and its geographic structure is weak (Souza et al., 2008). Beginning in 2007, individuals collected from bean seeds in the cities of Poços de Caldas and Alfenas, Minas Gerais, Brazil, were periodically incorporated into the stock population to avoid inbreeding negative effects. The stock population was maintained in our laboratory on bean seeds at 29 ± 2°C and 70 ± 5 % relative humidity in the dark. Additional information on the biology of this insect can be obtained from Bondar (1937), Teixeira et al. (2009), Teixeira and Gris (2011), and Teixeira and Zucoloto (2012).

We selected individuals to use chickpea for oviposition and development by randomly taking three sub-populations of the beetle stock population and putting them on *C. arietinum* seeds, and three other sub-populations were maintained on bean seeds. All seeds were previously stored at -10°C for at least 24 h to eliminate possible previous infestation. Each replicate was set up with 1 kg of seeds (~4,000) and ~1,000 founding adult individuals (1-6 days old). The populations were maintained on seeds obtained from a local commercial market (which commercializes pulse seeds freshly collected from the field) within 1.5 L (13 × 15 × 15 cm) plastic pots with perforated walls and lids. The experiment was run at 29 ± 2°C and 70 ± 5% relative humidity in the dark.

Since not all individuals of each pot emerge at the same time, all the emerged individuals (~1,000) in the third day of adult emergence from bean seeds were transferred to new pots with new seeds (there was no generation overlap). This was performed until reaching the 24^th^ generation of selection. As expected (Teixeira et al., 2008), the F1 on *C. arietinum* resulted in low adult emergence (~10 %). Thus, at the second or third day of the oviposition period of around ~9 days (high oviposition levels), only approximately 100 individuals of each replicate were transferred to a new pot to form the F2 generation. Increasing emergence percentage was observed during the next generations.

### RNA sequencing and transcriptome data analysis

RNA of equal concentration from the bean population and chickpea population at the 10^th^ generation of selection was used for RNA-sequencing using the Illumina platform (Genome Analyzer II, Life Sciences) at the Laboratório Multiusuário de Sequenciamento em Larga Escala e Expressão Gênica, Depto. de Tecnologia FCAV - UNESP - Jaboticabal, São Paulo, Brazil (http://bit.ly/facility-fcav). Since a genome is not yet available for *Z. subfasciatus*, we chose to sequence RNA representing a broad lifetime lapse, allowing us to get molecular information on key events of the reproductive cycle of the species in addition to that regarding the influence of host type use. Therefore, we combined RNA from different individuals into one single pool of RNA, for each treatment. RNA was extracted from two whole body females at each of the following developmental stages: late pharate-adult phase and 6 h, 24 h, and 72 h of adult life (three samples of this group of beetles were also used in qPCR assays for estimating the transcription levels of the selected genes *PGA*, *egl*, and *ACP-20*). Total RNA was extracted using TRIzol (Life Technologies), following the manufacturer’s protocol, as described previously (Mello et al., 2014). All sampled adult females were exposed to seeds and males. Libraries were prepared using the TruSeq RNA™ Sample Preparation kit (Illumina), for paired-end sequencing (2x75 bp).

Sequencing data was “gently trimmed” (MacManes, 2014) using TrimGalore with the following settings: ‘trim_galore --paired --retain_unpaired --phred33 --length 36 -q 5 --stringency 1 -e 0.1 ${i}R1_001.fastq.gz ${i}R2_001.fastq.gz’. Next, we assembled contigs using Trinity (v 2.4.0), following its standard settings for *de novo* assembly (Grabherr et al., 2011; Haas et al., 2013). We evaluated transcriptome completeness using BUSCO (v 5.5.0; Manni et al., 2021) and its insecta_odb10 database. We evaluated the percentage of sample alignment to *de novo* assembled transcripts using Bowtie2. Trinity (v 2.4.0) was also used to calculate quality metrics useful for evaluating success in reconstructing transcripts, such as N50, and Ex90N50. Trinity defines unigenes as the longest transcript isoform. Using Salmon (Patro et al., 2017), we estimated counts for each sample (number of reads and TPM), and next we estimated transcript and gene expression using the cross-sample normalization (Trimmed Means of M-values) script in Trinity. 

Trinotate (https://trinotate.github.io), (v 3.0.1) was used to identify coding regions (TransDecoder (Haas et al., 2013)) and annotate unigenes, transcripts and predicted proteins using the following databases: SwissProt, NCBI (nt/nr), EggNOG, and KEGG. Trinity typically identifies a greater number of transcripts (“isoforms”) and unigenes than the biologically expected number based on closely-related organisms with known genomes. Therefore, we filtered our count data to contain only transcripts and unigenes where coding regions (CDS) were identified by Transdecoder (minimum ORF length 100 amino acids). This strategy is based on the method adopted by Immonen et al. (2017). All downstream data analysis is based only on unigenes and transcripts that contain predicted coding regions.

Expression differences between and within samples were visualized by Principal Components Analysis and heatmaps. For each sample we also created a visual representation of their functional profile by counting the number of genes classified in Clusters of Orthologous Groups (COG).

We identified differentially expressed unigenes (“DEUs”) in pairwise comparisons between both samples. Gene expression analysis without replicates cannot exclude sample idiosyncrasies. We are confident, however, that the steps we took to design this study assure that results are reliable, such as: (1) samples are coming from a population with relatively homogeneous genetic background , (2) each sample contains RNA from multiple individuals, thus diluting any inter-individual variation; (3) running a qPCR experiment to further validate our results with greater sample size, and (4) we use methods accepted for gene expression analysis without replicates, which is an accepted design due to the relatively high cost of RNA-seq. Specifically, we used EdgeR’s approach for analysis without replicates by setting dispersion to 0.1, the recommended value for samples coming from a population with relatively homogeneous genetic background. We built a heatmap to visualize expression patterns for DEUs with an FDR-corrected p-value < 0.001 and fold change equals or greater than 4. To further investigate the expression profile in each sample, we hierarchically clustered DEUs according to expression values, and partitioned gene clusters using a percentage (60 %) of the height of the hierarchical tree. This task is accomplished in Trinity by using the function define_clusters_by_cutting_tree.pl. This command generates tables listing differentially expressed genes in each sample, for each cluster. In R, we cross-referenced these lists of genes with annotation from Trinotate, and selected genes associated with vitellogenic and olfactory functions for validation using qPCR.

For enrichment analysis, we used the GOSeq R package (Young et al., 2010) and Trinity scripts. For this analysis, we selected a subset of differentially expressed genes, with an FDR p-value smaller than 0.001 and a minimal logFC value of 2. The data that support the findings of this study are available at Sequence Read Archive (SRA, NCBI, http://www.ncbi.nlm.nih.gov/sra), under the Accession Number PRJNA798759 and from the corresponding author upon request.

### RNA extraction and RT-qPCR assays

For the transcript levels quantification assays by qPCR of the vitellogenic and olfactory genes, total RNA was extracted separately from pools of 36 heads (olfactory genes) and 12 abdomens + thoraces (vitellogenic genes) of individuals (24 h of adult life) of the different generations of selection using TRIzol (Life Technologies), following the manufacturer’s protocol, as described previously (Barchuk et al., 2004,^2007^). First strand cDNA was synthesized by reverse transcription from 1 (for olfactory genes) or 2.5 (for vitellogenic genes and *PGA, egl,* and *ACP-20*) μg of RNA with SuperScript II Reverse Transcriptase (Life Technologies) and an oligo(dT)_12-18_primer (Life Technologies). Comparative analyses of transcript levels were performed by Real Time quantitative PCR (qPCR) using a 7500 Real-Time PCR System (Applied Biosystems). Amplifications were carried out in 10 μL reaction mixtures, each containing 5 μL of SYBR^®^Green Master Mix 2× (Applied Biosystems), 0.4 μL of a 10 mM stock solution of each of the gene-specific forward and reverse primers (Table S1), and 0.5 μL of first-strand cDNA diluted 1:4 in ultrapure water. Reaction conditions were 50°C for 2 min, 95°C for 10 min, followed by 40 cycles of 95°C for 15 s and 60°C for 1 min. Three biological replicates were run in three technical replicates each. Relative quantities of the studied gene transcripts were calculated using the comparative Ct method (Applied Biosystems, User bulletin#2; Pfaffl, 2001; Livak and Schmittgen, 2001). Statistical analyses were carried out with Jandel SigmaStat 3.1 software (Jandel Corporation, San Rafael, CA, USA). Difference in transcript levels of the selected differentially expressed unigenes between samples was estimated by T test (*p* = 0.05) and that of vitellogenesis and olfaction genes between generations was estimated by two-way ANOVA (*p* ≤ 0.01) and Holm-Sidak.

Three genes were initially tested for their potential to be used as reference in qPCR assays: *ef1-α*,*rpl32,* and 18S RNA. The efficiency and stability of the respective pair of primers were tested in cDNA samples from males, females maintained on bean and females selected for the use of chickpea through generations (15 different types of samples). The Ct values obtained when testing the 18S gene were found to be too high, thus, the following testing was conducted only with *ef1-α* and*rpl32*. Stability analysis using Bestkeeper software (Pfaffl et al., 2004) revealed *rpl32* gene was more suitable for our samples (Tables S1-2), so that, it was used as reference in downstream assays.

## Results

### Genome-wide transcriptional profile of Bean and Chickpea populations of *Zabrotes subfasciatus*

The RNA library sequencing yielded 11,589,470 reads from the bean population library and 14,368,038 reads from the chickpea population library, from which 6,902,304 reads and 8,168,915 read aligned to genes, respectively (Table 1). After contig assembly and filtering for coding transcripts, we obtained 13,185 unigenes (longest transcript) and 20,827 transcripts (Table 2). Our assembly obtained a 96.2 % BUSCO score for completeness (Table 3). We found 400 unigenes and 1,202 transcripts differentially expressed between chickpea and bean populations (FDR *p* < 0.001; Tables S3 and S4). The transcriptional data are available at the Sequence Read Archive (SRA, NCBI, http://www.ncbi.nlm.nih.gov/sra), under the Accession Number PRJNA798759.

**Table 1 -:** Sample statistics on the transcriptomic analysis of the female populations of the bean beetle *Zabrotes subfasciatus* after 10 generation of selection on chickpea.

Raw assembly	Bean	Chickpea
Total paired-reads	11,589,470	14,368,038
Bowtie2 alignment rate	97.62 %	97.82 %
Aligned concordantly one time	5,597,586 (48.30 %)	6,751,501 (46.99 %)
Aligned concordantly >1 times	5,592,028 (48.25 %)	7,151,001 (49.77 %)
Reads aligned to genes (estimated counts)	11,152,198	13,870,240
Filtered Assembly		
Bowtie2 alignment rate	58.06 %	54.65 %
Aligned concordantly one time	3876721 (33.47 %)	4561673 (31.76 %)
Aligned concordantly >1 times	2444328 (21.10 %)	2773373 (19.31 %)
Reads aligned to genes (estimated counts)	6902304	8168915

**Table 2 -:** Assembly statistics on the transcriptomic analysis of the female populations of the bean beetle *Zabrotes subfasciatus* after 10 generation of selection on chickpea.

Trinity raw assembly stats	
“Genes”	41415
Transcripts	53316
% GC	40.15
N50	3222
N50 (longest isoform)	1872
Median contig length	492
Average contig length	1187.01
Median contig length (longest isoform)	392
Average contig length (longest isoform)	906.78
Trinity filtered assembly stats	
“Genes”	13185
Transcripts	20827
% GC	41.58
N50	3245
N50 (longest isoform)	2702
Median contig length	1636
Average contig length	2255.23
Median contig length (longest isoform)	1413
Average contig length (longest isoform)	1896

**Table 3 -:** Summary of BUSCO score results obtained for the *Zabrotes subfasciatus de novo* transcriptome assembly.

Number of BUSCOs	Type	Percentage of total
1314	Complete BUSCOs	96.2
929	Complete and single-copy BUSCOs	68
385	Complete and duplicated BUSCOs	28.2
17	Fragmented BUSCOs	1.2
36	Missing BUSCOs	2.6
1367	Total BUSCO groups searched	100

**Figure 1 -:**
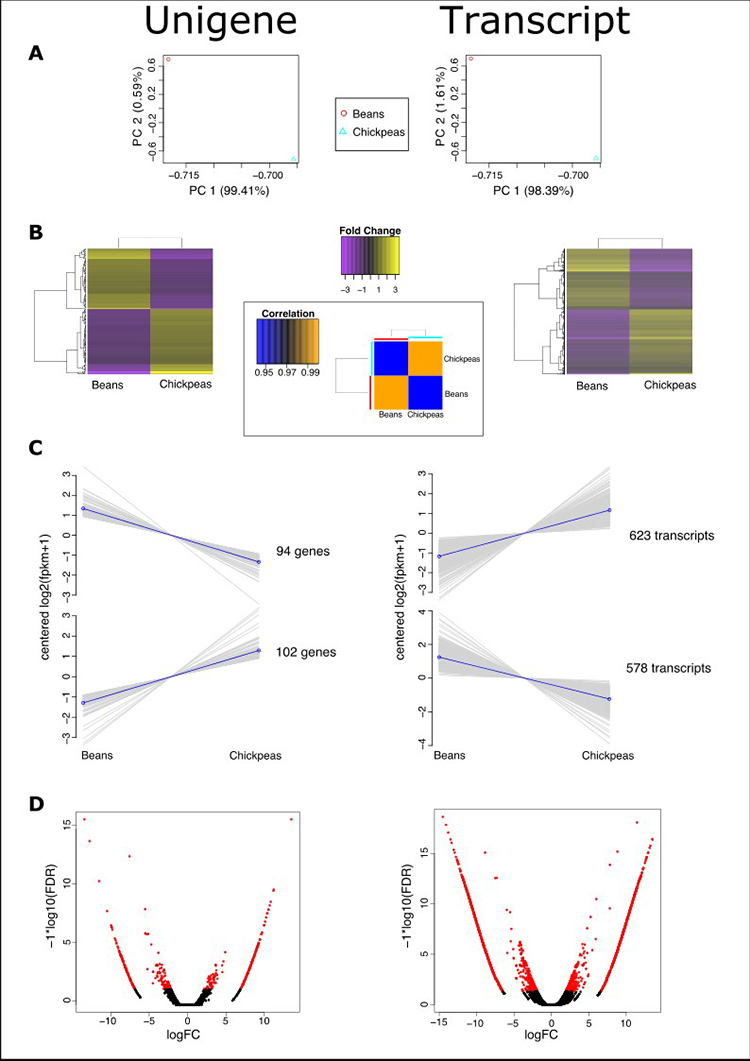
RNA-seq analysis summary for *Zabrotes subfasciatus*. Left, results using unigenes; Right, transcripts. A) Principal Components Analysis of log2 transformed counts (CPM) of TMM normalized values of expression. B) Left/Right: Heatmap of differentially expressed unigenes (FDR p<0.001) in bean and chickpea samples, for values with at least 4-fold expression change (TMM-normalized counts). Center: Heatmap of Pearson correlation values for expression values between samples (TMM-normalized counts, converted to CPM and log transformed). C) Groups of genes/transcripts with similar expression profile as depicted in the top 60 % height of the hierarchical clustering dendogram shown to the left of each heatmap in (B). D) Volcano plot representing unigenes/transcripts fold-change (log) by FDR-corrected p-values; differentially expressed values depicted in red.

PCA analysis of log2 counts of normalized expression values revealed that Bean and Chickpea beetle populations feature singular transcriptomic landscapes, when considering either unigenes (PC 99.41 %) or transcript variants (PC 98.39 %; Figure 1 A ). This was further shown after clustering the differentially expressed unigenes/variants (FDR p<0.001; values ≥ 4-fold expression change, TMM-normalized counts) and depicting them in heatmaps (Figure 1 B ). For the set of unigenes we were able to sample, about half of the expression was observed to be either up- or down-regulated in the chickpea population, including transcript variants, as shown in detail in Figure 1 C . The last graphs present the comparative expression values of the top 60 % unigenes (94 upregulated and 102 downregulated) and variants (623 upregulated and 578 downregulated). The higher number of differentially expressed transcript variants between populations depicted in Figure 1C is further visualized in the volcano plots of Figure 1 D .

**Figure 2 -:**
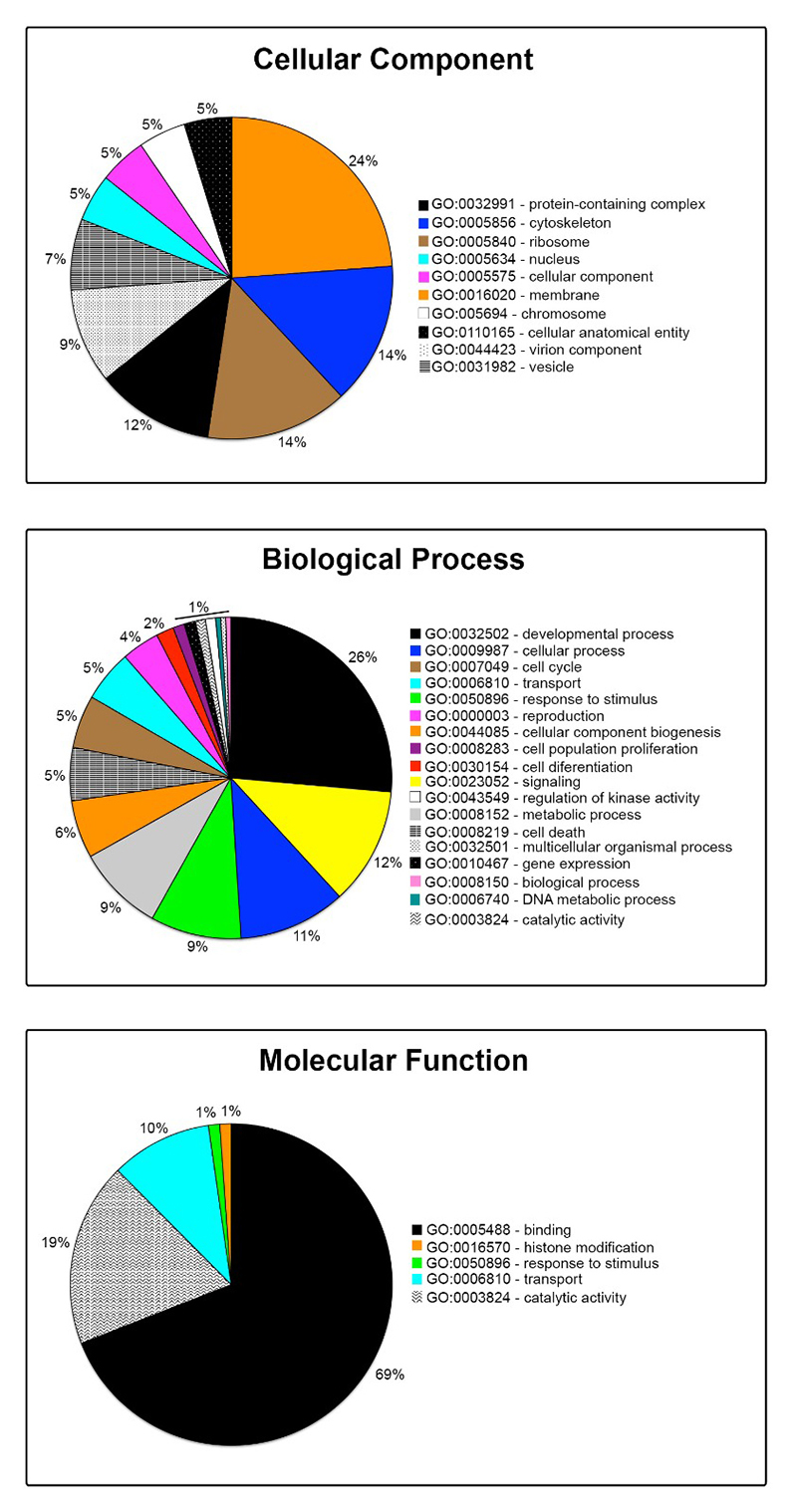
Gene Ontology enrichment analysis of the differentially expressed unigenes (DEUs) between Bean and Chickpea populations of *Zabrotes subfasciatus*. The GO terms are listed on the right of the graphs of each category (*p* < 0.001; logFC ≤ 2).

The differentially expressed genes between the studied populations of beetles (*p* < 0.001; logFC ≤ 2) are enriched in the main following Cellular Components terms: membrane (24 %), cytoskeleton and ribosome (14 % each), and protein-containing complex (12 %) (Figure 2). Among the overrepresented Biological Processes terms are the main following: Developmental processes (26 %), signalling (12 %), cellular processes (11 %), and response to stimulus and metabolic processes (9 % each) (Figure 2). Among the main overrepresented Molecular Function terms are the following: binding (protein or nucleotide, 69 %), catalytic activity (19 %), and transport (10 %) (Figure 2).

**Figure 3 -:**
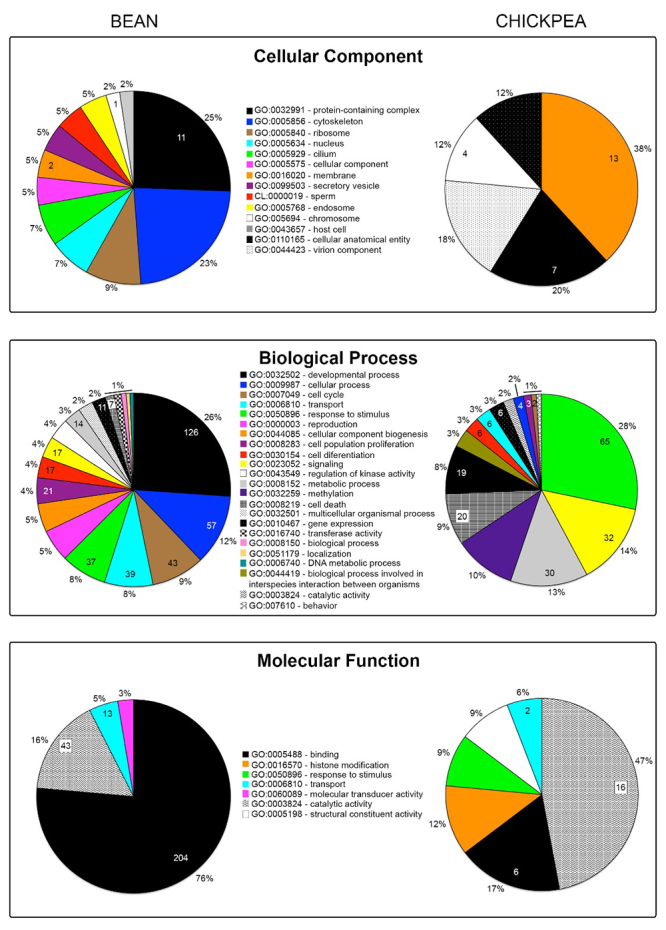
Gene Ontology enrichment analysis of the upregulated genes in the Bean and Chickpea populations of *Zabrotes subfasciatus*, respectively. The GO terms are listed between the graphs of each category. The percentage of each term is presented out of the graph and the absolute number of genes classified within a specific term is shown within the graph (*p* < 0.001; logFC ≤ 2).

GO enrichment analysis of the upregulated genes (*p* < 0.001; logFC ≤ 2) further validated that Bean and Chickpea beetle populations are characterized by singular transcriptomic landscapes. The Bean population features the overrepresentation of the following main Cellular Components terms: protein-containing complex (25 %), cytoskeleton (23 %), and ribosome (9 %); the Chickpea population, however, showed the following enriched terms: membrane (38 %), protein-containing complex (20 %), and cellular anatomical entity (12 %) (Figure 3). Among the Biological Process terms overrepresented in the Bean population were developmental process (26 %), cellular process (12 %), cell cycle (9 %), and response to stimulus (8 %); however, in the Chickpea population were response to stimulus (28 %), signalling (14 %), metabolic process (13 %), methylation (10 %), cell death (9 %), and developmental process (8 %) (Figure 3). Finally, the Bean population features the overrepresentation of the following main Molecular Function terms: binding (protein or nucleotide, 76 %), catalytic activity (16 %), and transport (5 %); the Chickpea population, on the other hand, shows the following main overrepresented terms: catalytic activity (46 %; e.g. P450 and decarboxylase enzymes), binding (17 %), histone modification (12 %), and response to stimulus (9 %) (Figure 3, Molecular Function).

### Transcriptional profile of selected differentially expressed unigenes (DEUs) between Bean and Chickpea populations of *Zabrotes subfasciatus*

**Figure 4 -:**
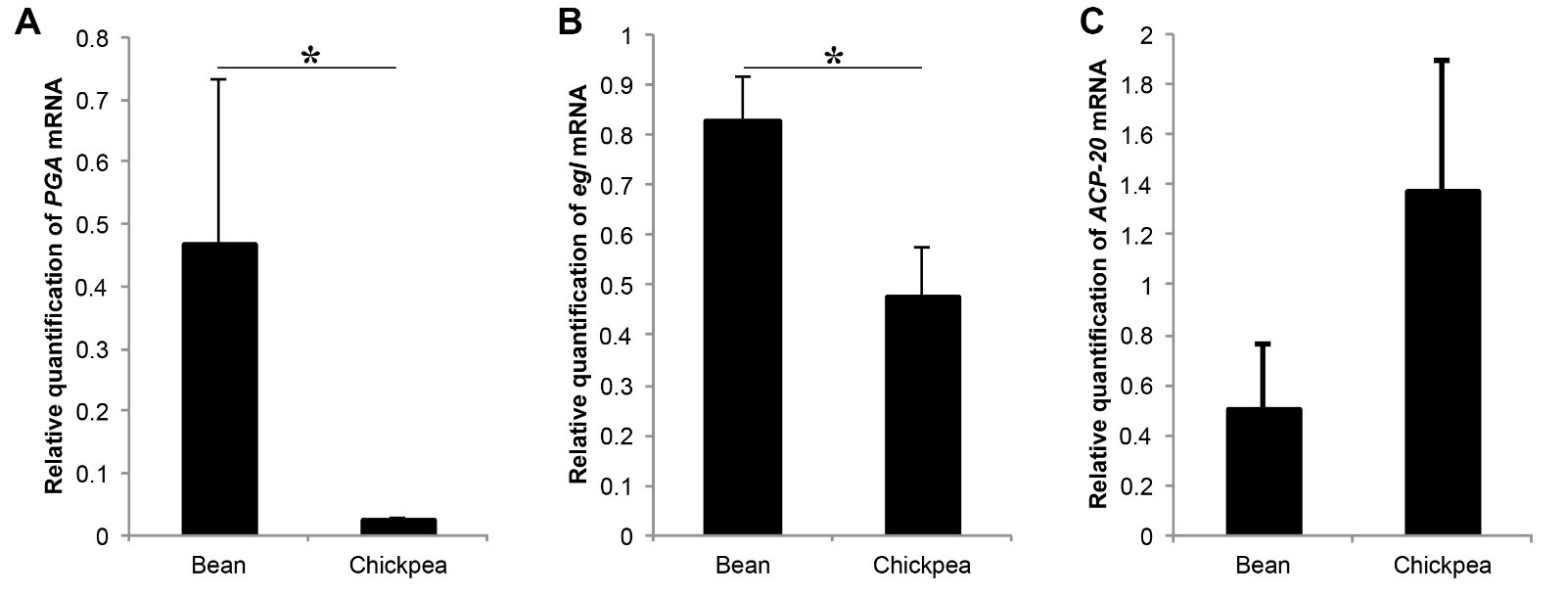
Transcript levels of selected differentially expressed unigenes (DEUs) between Bean and Chickpea populations of *Zabrotes subfasciatus*. A) *Polygalacturonase* (*PGA*). B) *Egalitarian* (*egl*). C) *Adult-specific cuticular protein ACP-20* (*ACP-20*). RNA samples consisted of three pools obtained from entire individuals after the selection experiment (late pharate-adult phase, 6 h, 24 h, and 72 h of adult life). Transcript levels were measured by qPCR and are presented as means + S.E.M. of three biological replicates, each run as three technical replicates. The relative expression was calculated using the ΔΔCt method with efficiency correction and a control sample for calibration (Pfaffl, 2001). Asterisks indicate statistical difference. T test *p* = 0.05.

Our tests on the suitability of genes as reference for gene expression analysis showed *rpl32* gene as being more appropriate for our samples (Tables S1-2). Thus, we used this gene as reference in both qPCR assays for estimating gene transcription levels. We first tested the transcript levels of three DEUs in RNA samples of individuals of the same experimental groups used for RNA sequencing (after 10 generations of selection): *Adult-specific cuticular protein ACP-20* (*ACP-20*), *egalitarian* (*egl*), and *polygalacturonase* (*PGA*). These genes were chosen (among those for which we could reconstruct transcripts with complete coding sequences) because of their known key participation in critical life-history traits development (see Discussion). Their transcript patterns can be seen in Figure 4: *PGA* and *egl* were found upregulated in the bean population (*p* = 0.05). Interestingly, *PGA* transcript levels were more than 4 times higher in the bean population beetles than in the chickpea population. *ACP-20* was found upregulated in the chickpea population (though not significantly). The transcription pattern of all three genes matches that obtained by RNA-Seq.

### Transcriptional response dynamics of vitellogenic and olfactory genes to host shift in *Zabrotes subfasciatus*

**Figure 5 -:**
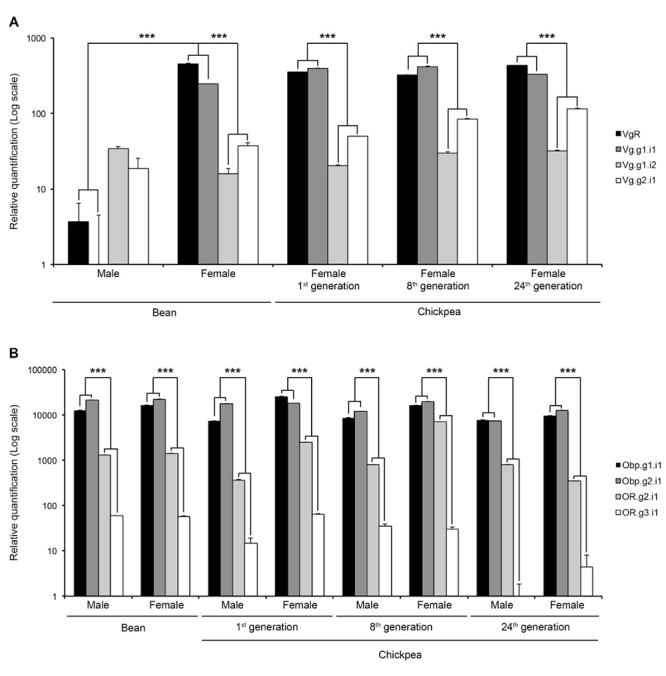
Transcriptional profile of genes related to vitellogenesis and olfaction in Bean and Chickpea populations of *Zabrotes subfasciatus -* clustered by treatment. A) Vitellogenic genes transcript levels (in abdomens plus thoraces). B) Olfactory genes transcript levels (in heads). Transcript levels were measured by qPCR and are presented as means + S.E.M. of three biological replicates, each run as three technical replicates. The relative expression was calculated using the comparative Ct method (Applied Biosystems, User bulletin#2 and Livak and Schmittgen, 2001). Data from males and females on bean were compared using t test (*p* ≤ 0.001), data within each generation were analysed by one-way ANOVA (*p* ≤ 0.001), data from males and females on chickpea and males and females on bean and those of different generations on chickpea were analysed by two-way ANOVA. Holm-Sidak method was used for all pairwise multiple comparisons. Asterisks indicate statistical difference.

**Figure 6 -:**
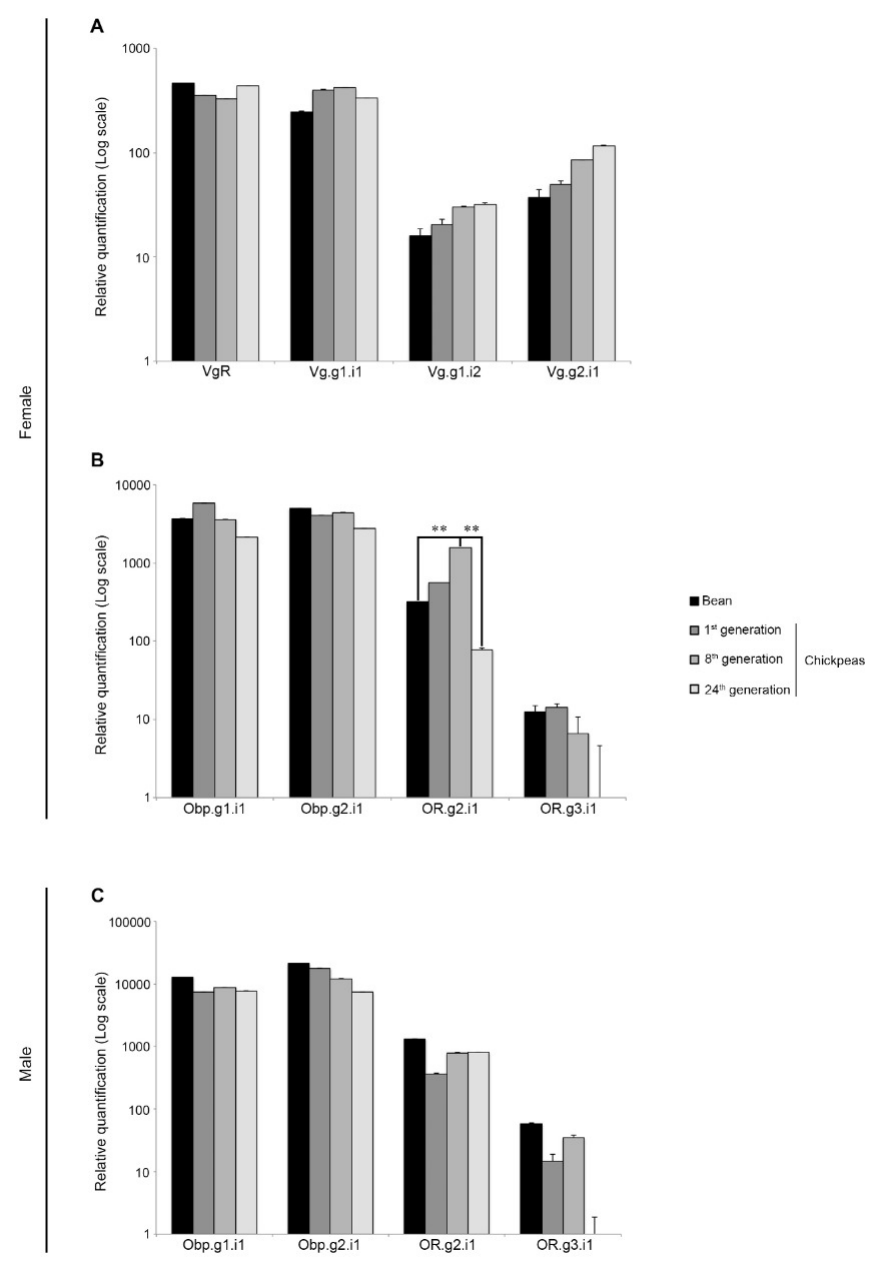
Transcriptional profile of genes related to vitellogenesis and olfaction in Bean and Chickpea populations of *Zabrotes subfasciatus* - clustered by genes. A) Transcript levels of vitellogenic genes in females (abdomens plus thoraces). B) Transcript levels of olfactory genes in females (heads). C) Transcript levels of olfactory genes in males (heads). The relative expression was calculated using the comparative Ct method (Applied Biosystems, User bulletin#2 and Livak and Schmittgen, 2001). Data from each gene on bean and during generations were compared by one-way ANOVA (*p* ≤ 0.01). Holm-Sidak method was used for all pairwise multiple comparisons. Asterisks indicate statistical difference.

Aiming at testing the beetles’ molecular response to the selection for the use of a new host, we then determined the transcript levels of vitellogenic (Vitellogenin Receptor VgR, and Vitellogenins Vg.g1.i1, Vg.g1.i2, and Vg.g2.i1) and olfactory unigenes (Odorant binding proteins Obp.g1.i1, Obp.g2.i1, and Odorant Receptors OR.g2.i1, and OR.g3.i1). The tested unigenes within these groups were those for which we could reconstruct transcripts with complete coding sequences (g= gene; i= isoform). The respective genes are known to be directly involved with reproductive capacity and odorant recognition, key life-history traits and determinants of Darwinian fitness within the context of insect-plant interaction (Wu et al., 2021; Simon et al., 2015). Vitellogenic genes expression was determined in abdomens + thoraces and olfactory genes expression was determined in heads of males and females belonging the stock population and to the generations 1^st^, 8^th^, and 24^th^ on chickpea. We first showed that individuals of all groups (sex and experimental condition) express all the tested genes (Figure 5). In spite of male samples being included as reference (vitellogenic genes are characteristic of the reproductive physiology of females), maybe one of the most interesting findings was that males express vitellogenic genes (*Vg* and its receptor, *VgR*), with Vg.g1.i2 and Vg.g2.i1 in similar levels to those registered in females (Figure 5 A ). Vg.g1.i1 and VgR transcript levels, though, were found in lower levels in males than in females of all groups. In all conditions, females showed higher levels of transcription of the pair VgR and Vg.g1.i1 than the other tested unigenes. The second group of genes (olfactory genes) showed a transcription pattern shared by males and females in the different conditions: Obp.g1.i1 and Obp.g2.i1 express in higher levels than the studied receptors, OR.g2.i1 and OR.g3.i1 (Figure 5 B ). The transcriptional dynamics of the analysed genes showed alterations during the generations of selection (Figure 6). The analysis showed that both Vg.g1.i2 and Vg.g2.i1 seem to increase their transcription levels over generations of selection (though not significantly). Moreover, one of the olfactory unigenes, OR.g2.i1, clearly varies in expression levels among experimental groups, i.e., OR.g2.i1 shows higher expression after 8 generation of selection on chickpea than in females maintained in bean. At the 24^th^ generation, though, expression is back to levels comparable to those of the initial population (Figure 6 B ).

## Discussion

### On the genome-wide transcriptional signature of host shift in *Zabrotes subfasciatus*

Our results are congruent with the hypothesis that early steps of *Z. subfasciatus* adaptation to using a new host are underpinned by a shift in its gene expression landscape. Our data shows that after 10 generations of selection for the use of *C. arietinum*, *Z. subfasciatus* females show striking different transcription patterns compared to those kept on bean, with each population of beetles differentially regulating distinct subsets of genes. Interestingly, in addition to showing different expression levels of certain groups of genes, the populations showed differential expression of transcript variants, which may result from differential use of transcription start sites or alternative splicing (Haberle and Stark, 2018; Gehring and Roignant, 2021). Isoforms are known to vary drastically in function, and even have opposite functions, as those involved in the sex determining pathway (Salz, 2011) and the downstream events promoted by juvenile hormone through *taiman* (Liu et al., 2018). Therefore, alternative variants of a gene may help insects adapt to a new host by coding different proteins that can equip them with functions that could bring fitness to levels compatible with a host switch.

The factors that influence the initial stages of host choice for insect oviposition include ones that act at long distances and promote alighting. After this first contact, females usually evaluate the host and are influenced by volatile, gustatory or tactile cues (Knolhoff and Heckel, 2014; unpublished material from our laboratory). After choosing an oviposition site, seed beetle females lay eggs on the seed or pod surface and newly emerged larvae burrow into the seed, where they feed and undergo larval and pupal development (Howe and Currie, 1964). Thus, most genes found here as differentially regulated are mainly those which would be expected to sequentially play a role in inset adaptation to a new site for oviposition and development: response to stimulus, signalling, and developmental processes. Interestingly, unlike the Bean population, Chickpea population showed the upregulation of genes whose proteins are involved in the catalysis of methylation events (catalytic activity is the term with the highest number of upregulated genes in this population), particularly histone modification (e.g. histone-arginine methyltransferase, histone-lysine N-methyltransferase). It is known that environment can change the phenotype and alter phenotypic variation through epigenetic mechanisms, which seems now to be essential for evolution (Mogilicherla and Roy, 2023). One such epigenetic mechanism requires the participation of histone methyltransferases (which add methyl groups to specific histone amino acids), for which there are about 30 genes in insects (Palli, 2021). Histone methylation may thus constitute a key player in the adaptation to the use of a novel host in *Z. subfasciatus*, which deserves further investigation, along with the participation of cell death genes (encoding proteins involved in regulating apoptosis, e.g. POZ, runt-related transcription factor, and programmed cell death proteins), also upregulated in the Chickpea population.

### On the transcriptional patterns of life-history development genes during the artificial selection for the use of an alternative host in *Zabrotes subfasciatus*

We used qPCR to perform an in-depth analysis of the transcriptional response of key genes to the selection of *Z. subfasciatus* populations for the use of an alternative host. The genes of the first subset were already revealed by the RNA-Seq assay as differentially expressed between populations. One of them, *PGA*, is known to code for an enzyme responsible for digesting the galacturonic acid rich backbone of the pectin matrix of cell walls of growing plant parts (Mohnen, 2008). The gene, initially thought to be restricted to plants, bacteria, and fungi, was also found in diverse animal species, to where it has arrived through horizontal gene transfer (Kirsch et al., 2014). Interestingly, here we show *PGA* expression in the seed beetle *Z. subfasciatus* and that its expression is higher in beetles from the bean population than in those selected for the use of the alternative host, chickpea. PGA secreted by the leaf beetle *Phaedon cochleariae* promotes the efficient release of nutrients by its host, the Chinese cabbage (*Brassica rapa*), thus increasing the insect fitness (Kirsch *et al.*, 2022). As part of this insect-plant arm-race, host plants of another Coleoptera, the Bruchinae *C. maculatus*, were reported to express PGA inhibitors to defend themselves against these beetles (Rathnayaka Gamage et al., 2022). The higher *PGA* expression in *Z. subfasciatus* from the bean population may constitute a key molecular adaptation to this most preferred host. Since our qPCR assays were conducted using RNA pools of individuals at pharate-adult developmental phase and at different ages of adult life, the high levels of *PGA* mRNA may represent signs of seed tissues digestion after the larval stage (adult feeding on artificial diets by *Z. subfasciatus* was already reported, Corrêa et al., 2020). Future studies should focus on the mechanism regulating beetles *PGA* expression and expression during development within chickpea seeds.

In *Drosophila*, Egl, interacting with a few other proteins, acts as an adaptor between a cargo (e.g. mRNA molecules) and dynein (Nashchekin and St Johnston, 2009; Baker et al., 2021). By doing this, it fundamentally contributes with oocyte determination and dorsal-ventral patterning (Deng and Lin, 2001). In our work, we show that *egl* transcription levels are higher in the bean population than in the chickpea population, likely reflecting the role of this gene in assuring high levels of reproductive output to beetles growing on this host. Moreover, gain-of-function experiments have suggested Egl may play a role in the salivary glands development (Maybeck and Röper, 2009).Salivary gland secretions might play key roles in permitting beetles to exploit their host seeds, e.g., helping with the initial digestion of seed tissues, which can represent an additional biological system in which this protein may differently contribute with *Z. subfasciatus* host shift.

*ACP-20*, which codes for a member of adult-specific cuticular proteins, was found upregulated in the chickpea population. Interestingly, cuticular protein genes are commonly found differentially expressed in studies tackling insect adaptation to alternative hosts through transcriptomics (Celorio-Mancera *et al.*, 2013; Hoang et al., 2015; Birnbaum and Abbot, 2020). It’s been suggested that this finding highlights the role of cuticle restructuring associated with populations adaptation to alternative hosts (Birnbaum and Abbot, 2020). It remains to be determined, though, in which way varying levels of cuticle proteins can impinge alterations in adaptive values during the dynamics of host shift in phytophagous insects, particularly in those beetles using chickpea seeds.

The genes of the second subset, for which we performed qPCR assays to get insight into their transcriptional behaviour between sexes and during the selection experiments, are known to be involved in the development of two key life-history traits: vitellogenic and olfactory genes. These genes are involved in early steps of the oviposition substrate or mate pair recognition (olfactory genes) and ovary development/activation (vitellogenic genes). Here we refer as vitellogenic the genes encoding vitellogenin proteins (Vg1 and Vg2) and its putative receptor (VgR). Vitellogenin (Vg) was initially thought to be the egg yolk precursor protein, though recently several reports revealed its pleiotropic functions (Salmela et al., 2022). To accomplish its canonical function, Vg molecules are secreted mainly by fat body cells (or follicle cells, nurse cells, and hemocytes) and taken up by oocytes via receptor-mediated (VgR) endocytosis (Wu et al., 2021). The expression of Vg1.i2 and Vg2.i1 gene variants that we observed in males at similar levels to those recorded in females, and the higher expression of Vg1.i1 and VgR transcripts in females, both suggest that there is a differential use of *Vg* genes and transcript variants in *Z. subfasciatus*. The Vg1.i1 variant seems to harbour the canonical reproductive function, along with the VgR, promoting vitellogenesis. The other variants might be involved in non-canonical functions, important for both sexes. Moreover, the higher levels of Vg expression in males relative to VgR expression suggest that in males Vg acts as an extracellular protein performing a yet unknown function. Vitellogenin, whose gene expression is under hormonal control (Mello et al., 2019), has already been reported to have several functions in addition to its canonical vitellogenic one. For instance, planthopper *Laodelphax striatellus* salivary glands “injects” vitellogenin into host plants, which acts as an effector impairing plant defence (Ji et al., 2021). Vitellogenin also acts as an immunomodulator, antioxidant, and, as recently suggested, as a regulatory nuclear protein (Salmela *et al.*, 2022). *Vg* expression in males was also reported for other species (*L. striatellus*, Huo et al., 2018; honeybee drones Piulachs et al., 2003), though with no differential use of possible isoforms. The apparent increase of Vg.g1.i2 and Vg.g2.i1 transcription levels in females over generations of selection on chickpea suggest their involvement in functions allowing the beetle’s adaptation to the new host.

In insects, the olfactory system detects odorants, essential for feeding, mating, and avoiding hostile environments and toxic substances, and its rapid evolution suggests it is involved in fast adaptation to changing environments (Zhou, 2010). Since most odorants are small hydrophobic molecules (odorants and pheromones), they enter the antenna or palp sensillum and need to be transported by odorant-binding proteins to odorant receptors (ORs), to which they bind directly or complexed with an Obp (Zhou, 2010; Mika and Benton, 2021). Among the reported large families of odorant-binding and odorant receptor proteins (Zhou, 2010; Zhang et al., 2017; Mitchell et al., 2020; Tanaka et al., 2022), we gained information on the transcriptional behaviour of two odorant-binding protein (Obp.g1.i1 and Obp.g2.i1) and two odorant receptor protein genes (OR.g2.i1 and OR.g3.i1). The observed similar transcription pattern of olfactory genes in females and males was surprising since in *Z. subfasciatus* there seems to exist a functional sexual diphenism regarding olfaction, with females mainly involved in host volatile organic compounds sensing and males in female-emitted pheromone sensing (unpublished material from our laboratory). Future gene annotation experiments on genomic DNA will allow for gain of information on the occasional existence of molecular diphenism regarding the olfactory system of *Z. subfasciatus* males and females.

We acknowledge that our study contains important caveats: our sample size is small, *Z. subfasciatus* lacks a reference genome, and our lack of replicates (for RNA-seq) warrants caution for the interpretation of our results. On the other hand, our qPCR results further extend the RNA-seq data, with an increased number of replicates. Moreover, our study provides fundamental information regarding gene and protein sequences that were previously unknown for this species. This information adds to an increasing nucleotide database that is of essence for comparative and evolutionary biology. In addition, the differences we found in gene expression are congruent with other plant-insect systems and can be used as basis for the design of follow-up studies.

Summarizing, our study provides evidence that the early steps of *Z. subfasciatus* adaptation to the use of a new host for oviposition and development are underpinned by a shift in its gene expression landscape, which involves the differential expression of distinct subsets of genes and a significant differential expression of transcript variants. Most of the differentially regulated genes between populations are those expected to allow key steps in using an alternative host: response to stimulus, signalling, and developmental processes genes. The population selected for the use of a new host shows the upregulation of methylation genes, particularly histone methylation, which may constitute a strategy for fine-tuning the insect global gene expression network leading *Z. subfasciatus* to the adaptation to the use of the novel host. We also suggest the existence of Vg variants (whose expression seems to vary during the selection) possible harbouring canonical reproductive function in females and other Vg variants performing extracellular unknown functions in males. Future genomic approaches will shed light on the nature of the proximate mechanisms involved in promoting the differential gene expression pattern seen between the beetle populations studied here.

## Supplementary material

The following online material is available for this article:

Table S1 - Characteristics of the primers used to test the transcriptional profile of selected differentially expressed unigenes (DEUs) between Bean and Chickpea populations of *Zabrotes subfasciatus*

Table S2 - Descriptive statistics of two reference genes for qPCR assays of *Zabrotes subfasciatus* samples (calculated via BestKeeper).

Table S3 - Differentially expressed unigenes (DEUs) between Bean and Chickpea populations of *Zabrotes subfasciatus*. Includes annotation, logFC, logCPM, and both P-value and FDR. All transcripts (isoforms and gene ids) were annotated by the Trinotate system.

Table S4 - Differentially expressed transcripts between Bean and Chickpea populations of *Zabrotes subfasciatus*. Includes annotation, logFC, logCPM, and both P-value and FDR. All transcripts (isoforms and gene ids) were annotated by the Trinotate system.

## References

[B1] Baker FC, Neiswender H, Veeranan-Karmegam R, Gonsalvez GB (2021). In vivo proximity biotin ligation identifies the interactome of Egalitarian, a Dynein cargo adaptor. Development.

[B2] Barchuk AR, Maleszka R, Simões ZLP (2004). Apis mellifera ultraspiracle: cDNA sequence and rapid up-regulation by juvenile hormone. Insect Mol Biol.

[B3] Barchuk AR, Cristino AS, Kucharski R, Costa LF, Simões ZLP, Maleszka R (2007). Molecular determinants of caste differentiation in the highly eusocial honeybee Apis mellifera. BMC Dev Biol.

[B4] Birnbaum SSL, Abbot P (2020). Gene expression and diet breadth in plant-feeding insects: Summarizing trends. Trends Ecol Evol.

[B5] Bondar G (1937). Notas biológicas sobre Bruchideos observados no Brasil. Arq Inst Biol Veg.

[B6] Cardona C, Hodges R, Farrel G (2004). Crop post-harvest: Science and technology.

[B7] MdlP Celorio-Mancera, Wheat CW, Vogel H (2013). Mechanisms of macroevolution: Polyphagous plasticity in butterfly larvae revealed by RNA-Seq. Mol Ecol.

[B8] Corrêa CP, Capizzani BC, Beijo LA, Ávila PM, Teixeira IRV, Barchuk AR (2020). Adult feeding and host type modulate the life history traits of the capital breeder Zabrotes subfasciatus. Physiol Entomol.

[B9] Corrêa CP, Parreiras SS, Beijo LA, Ávila PM, Teixeira IRV, Barchuk AR (2021). Life history trait response to ambient temperature and food availability variations in the bean weevil Zabrotes subfasciatus. Physiol Entomol.

[B10] Deng W, Lin H (2001). Asymmetric germ cell division and oocyte determination during Drosophila oogenesis. Int Rev Cytol.

[B11] Desroches P, Mandon N, Baehr JC, Huignard J (1997). Mediation of host-plant use by a glucoside in Callosobruchus maculatus F. (Coleoptera: Bruchidae). J Insect Physiol.

[B12] Ehrlich PR, Raven PH (1964). Butterflies and plants: A study in coevolution. Evolution.

[B13] Farrell BD (1998). “Inordinate Fondness” explained: Why are there so many beetles?. Science.

[B14] Forbes AA, Devine SN, Hippee AC, Tvedte ES, Ward AKG, Widmayer HA, Wilson CJ (2017). Revisiting the particular role of host shifts in initiating insect speciation. Evolution.

[B15] Gehring NH, Roignant J-Y (2021). Anything but ordinary - Emerging splicing mechanisms in eukaryotic gene regulation. Trends Gen.

[B16] Grabherr MG, Haas BJ, Yassour M, Levin JZ, Thompson DA, Amit I, Adiconis X, Fan L, Raychowdhury R, Zeng Q (2011). Full-length transcriptome assembly from RNA-Seq data without a reference genome. Nat Biotechnol.

[B17] Haas BJ, Papanicolaou A, Yassour M, Grabherr M, Blood PD, Bowden J, Couger MB, Eccles D, Li B, Lieber M (2013). De novo transcript sequence reconstruction from RNA-seq using the Trinity platform for reference generation and analysis. Nat Protoc.

[B18] Haberle V, Stark A (2018). Eukaryotic core promoters and the functional basis of transcription initiation. Nat Rev Mol Cell Biol.

[B19] Hoang K, Matzkin LM, Bono JM (2015). Transcriptional variation associated with cactus host plant adaptation in Drosophila mettleri populations. Mol Ecol.

[B20] Howe RN, Currie JE (1964). Some laboratory observations on the rates of development, mortality and oviposition of several species of Bruchidae breeding in stored pulses. Bull Entomol Res.

[B21] Huo Y, Yu Y, Chen L, Li Q, Zhang M, Song Z, Chen X, Fang R, Zhang L (2018). Insect tissue-specific vitellogenin facilitates transmission of plant virus. PLoS Pathog.

[B22] Immonen E, Sayadi A, Bayram H, Arnqvist G (2017). Mating changes sexually dimorphic gene expression in the seed beetle Callosobruchus maculatus. Genome Biol Evol.

[B23] Ji R, Fu J, Shi Y, Li J, Jing M, Wang L, Yang S, Tian T, Wang L, Ju J (2021). Vitellogenin from planthopper oral secretion acts as a novel effector to impair plant defenses. New Phytol.

[B24] Johnson CD, Polhill RM, Raven PH (1981). Advances in legume systematics 2.

[B25] Kingsolver JM (2004). Handbook of the Bruchidae of the United States and Canada (Insecta: Coleoptera). USDA Tech Bull.

[B26] Kirsch R, Gramzow L, Theißen G, Siegfried BD, Ffrench-Constant RH, Heckel DG, Pauchet Y (2014). Horizontal gene transfer and functional diversification of plant cell wall degrading polygalacturonases: Key events in the evolution of herbivory in beetles. Insect Biochem Mol Biol.

[B27] Kirsch R, Okamura Y, Haeger W, Vogel H, Kunert G, Pauchet Y (2022). Metabolic novelty originating from horizontal gene transfer is essential for leaf beetle survival. Proc Natl Acad Sci U S A.

[B28] Knolhoff LM, Heckel DG (2014). Behavioral assays for studies of host plant choice and adaptation in herbivorous insects. Annu Rev Entomol.

[B29] Langmüller AM, Schlötterer C (2020). Low concordance of short‐term and long‐term selection responses in experimental Drosophila populations. Molec Ecol.

[B30] Lawrence JF, Parker SP (1982). Synopsis and classification of living organisms.

[B31] Liu P, Fu X, Zhu J (2018). Juvenile hormone-regulated alternative splicing of the taiman gene primes the ecdysteroid response in adult mosquitoes. Proc Natl Acad Sci U S A.

[B32] Livak KJ, Schmittgen TD (2001). Analysis of relative gene expression data using real-time quantitative PCR and the 2-ΔΔCT method. Methods.

[B33] Lucas MH (1858). Spermophagus semifasicatus. Bull Ent In Annls Socent Ft.

[B34] MacManes M (2014). On the optimal trimming of high-throughput mRNA sequence data. Front Genet.

[B35] Manni M, Berkeley MR, Seppey M, Simão FA, Zdobnov EM (2021). BUSCO update: Novel and streamlined workflows along with broader and deeper phylogenetic coverage for scoring of eukaryotic, prokaryotic, and viral genomes. Mol Biol Evol.

[B36] Maybeck V, Röper K (2009). A targeted gain-of-function screen identifies genes affecting salivary gland morphogenesis/tubulogenesis in Drosophila. Genetics.

[B37] McKenna DD, Scully ED, Pauchet Y, Hoover K, Kirsch R, Geib SM, Mitchell RF, Waterhouse RM, Ahn SJ, Arsala D (2016). Genome of the Asian longhorned beetle (Anoplophora glabripennis), a globally significant invasive species, reveals key functional and evolutionary innovations at the beetle-plant interface. Genome Biol.

[B38] McKenna DD, Shin S, Ahrens D, Balke M, Beza-Beza C, Clarke DJ, Donath A, Escalona HE, Friedrich F, Letsch H (2019). The evolution and genomic basis of beetle diversity. Proc Natl Acad Sci U S A.

[B39] Meik J, Dobie P (1986). The ability of Zabrotes subfasciatus to attack cowpeas. Entomol Exp Appl.

[B40] Mello TR, Aleixo AC, Pinheiro DG, Nunes FM, Bitondi MM, Hartfelder K, Barchuk AR, Simões ZL (2014). Developmental regulation of ecdysone receptor (EcR) and EcR-controlled gene expression during pharate-adult development of honeybees (Apis mellifera). Front Genet.

[B41] Mello TRP, Aleixo AC, Pinheiro DG, Nunes FMF, Cristino AS, Bitondi MMG, Barchuk AR, Simões ZLP (2019). Hormonal control and target genes of ftz-f1 expression in the honeybee Apis mellifera: A positive loop linking juvenile hormone, ftz-f1, and vitellogenin. Insect Mol Biol.

[B42] Messina FJ, Jones JC, Mendenhall M, Muller A (2009). Genetic modification of host acceptance by a seed beetle, Callosobruchus Maculatus (Coleoptera: Bruchidae). Ann Entomol Soc Am.

[B43] Mika K, Benton R (2021). Olfactory receptor gene regulation in insects: Multiple mechanisms for singular expression. Front Neurosci.

[B44] Mitchell RF, Schneider TM, Schwartz AM, Andersson MN, McKenna DD (2020). The diversity and evolution of odorant receptors in beetles (Coleoptera). Insect Mol Biol.

[B45] Mogilicherla K, Roy A (2023). Epigenetic regulations as drivers of insecticide resistance and resilience to climate change in arthropod pests. Front Genet.

[B46] Mohnen D (2008). Pectin structure and biosynthesis. Curr Opin Plant Biol.

[B47] Nashchekin D, St Johnston D (2009). Egalitarian recruitment of localized mRNAs. Genes Dev.

[B48] Oppenheim SJ, Baker RH, Simon S, DeSalle R (2015). We can’t all be supermodels: The value of comparative transcriptomics to the study of non-model insects. Insect Mol Biol.

[B49] Palli SR (2021). Epigenetic regulation of post-embryonic development. Curr Opin Insect Sci.

[B50] Patro R, Duggal G, Love MI, Irizarry RA, Kingsford C (2017). Salmon provides fast and bias-aware quantification of transcript expression. Nat Methods.

[B51] Pfaffl MW (2001). A new mathematical model for relative quantification in real-time RT-PCR. Nucleic Acids Res.

[B52] Pfaffl MW, Tichopad A, Prgomet C, Neuvians TP (2004). Determination of stable housekeeping genes, differentially regulated target genes and sample integrity: BestKeeper--Excel-based tool using pair-wise correlations. Biotechnol Lett.

[B53] Piulachs MD, Guidugli KR, Barchuk AR, Cruz J, Simões ZL, Bellés X (2003). The vitellogenin of the honey bee, Apis mellifera: Structural analysis of the cDNA and expression studies. Insect Biochem Mol Biol.

[B54] Rathnayaka Gamage SI, Kaewwongwal A, Laosatit K, Yimram T, Lin Y, Chen X, Nakazono M, Somta P (2022). Tandemly duplicated genes encoding polygalacturonase inhibitors are associated with bruchid (Callosobruchus chinensis) resistance in moth bean (Vigna aconitifolia). Plant Sci.

[B55] Rêgo A, Messina FJ, Gompert Z (2019). Dynamics of genomic change during evolutionary rescue in the seed beetle Callosobruchus maculatus. Mol Ecol.

[B56] Rêgo A, Chaturvedi S, Springer A, Lish AM, Barton CL, Kapheim KM, Messina FJ, Gompert Z (2020). Combining experimental evolution and genomics to understand how seed beetles adapt to a marginal host plant. Genes (Basel).

[B57] Salmela H, Harwood GP, Münch D, Elsik CG, Herrero-Galán E, Vartiainen MK, Amdam GV (2022). Nuclear translocation of vitellogenin in the honey bee (Apis mellifera). Apidologie.

[B58] Salz HK (2011). Sex determination in insects: A binary decision based on alternative splicing. Curr Opin Genet Dev.

[B59] Schoch CL, Ciufo S, Domrachev M, Hotton CL, Kannan S, Khovanskaya R, Leipe D, Mcveigh R, O’Neill K, Robbertse B, Sharma S (2020). NCBI Taxonomy: A comprehensive update on curation, resources and tools. Database (Oxford).

[B60] Simon JC, d’Alençon E, Guy E, Jacquin-Joly E, Jaquiéry J, Nouhaud P, Peccoud J, Sugio A, Streiff R (2015). Genomics of adaptation to host-plants in herbivorous insects. Brief Funct Genomics.

[B61] Southgate BJ (1979). Biology of the Bruchidae. Ann Rev Entomol.

[B62] Souza GA, Carvalho MRO, Martins ER, Guedes RNC, Oliveira LO (2008). Genetic diversity estimated through ISSR markers in populations of Zabrotes subfasciatus. Pesq Agrope Bras.

[B63] Tanaka K, Shimomura K, Hosoi A, Sato Y, Oikawa Y, Seino Y, Kuribara T, Yajima S, Tomizawa M (2022). Antennal transcriptome analysis of chemosensory genes in the cowpea beetle, Callosobruchus maculatus (F.). PLoS One.

[B64] Teixeira IRV, Zucoloto FS (2003). Seed suitability and oviposition behaviour of wild and selected populations of Zabrotes subfasciatus (Boheman) (Coleoptera, Bruchidae) on different hosts. J Stored Prod Res.

[B65] Teixeira IRV, Barchuk AR, Zucoloto FS (2008). Host preference of the bean weevil Zabrotes subfasciatus. Insect Sci.

[B66] Teixeira IRV, Barchuk AR, Medeiros L, Zucoloto FS (2009). Females of the weevil Zabrotes subfasciatus manipulate the size and number of eggs according to the host seed availability. Physiol Entomol.

[B67] Teixeira IRV, Gris CF, Popescu E, Golubev I (2011). Beans: Nutrition, consumption and health.

[B68] Teixeira IRV, Zucoloto FS (2012). Intraspecific competition in Zabrotes subfasciatus: Physiological and behavioral adaptations to different amounts of host. Insect Sci.

[B69] Valencia G, Carlos A, Cardona Mejía C, Schoonhoven A van (1986). Main insect pests of stored beans and their control [tutorial unit].

[B70] Wu Z, Yang L, He Q, Zhou S (2021). Regulatory mechanisms of vitellogenesis in insects. Front Cell Dev Biol.

[B71] Young MD, Wakefield MJ, Smyth GK, Oshlack A (2010). Gene ontology analysis for RNA-seq: Accounting for selection bias. Genome Biol.

[B72] Zhang Y-N, Kang K, Xu L, Zhu X-Y, Qian J-L, Zhang Z-J, He P, Li X-M (2017). Deep sequencing of antennal transcriptome from Callosobruchus chinensis to characterize odorant binding protein and chemosensory protein genes. J Stored Prod Res.

[B73] Zhou JJ (2010). Odorant-binding proteins in insects. Vitam Horm.

