# Supplementary material for: Transcriptional signature of host shift in the seed beetle Zabrotes subfasciatus
Source: Genet Mol Biol. 2024 Feb 5;47(1):e20230148. doi: 10.1590/1678-4685-GMB-2023-0148 (PMC10851049; doi:10.1590/1678-4685-GMB-2023-0148)
Supplement: Table S1 - [file 1415-4757-GMB-47-01-e20230148-s1.pdf]

Genetics and Molecular Biology
https://doi.org/10.1590/1678-4685-GMB-2023-0148

Supplementary Material to "Transcriptional signature of host shift in the seed beetle *Zabrotes subfasciatus*"

Table S1 - Characteristics of the primers used to test the transcriptional profile of selected differentially expressed genes (DEGs) between Bean and Chickpea populations of *Zabrotes subfasciatus*.

Gene symbol and name	Primer sequence (5' → 3')	Amplicon size (bp)	TM (°C)	R^2	Slope	Efficiency (%)	Amplification factor
rpl32 (ribossomal protein L32)	Forward TACACAACGTGAGGGAACTG Reverse AGCATGTCCGTTCGTTACTC	146	60	0.98	-2.994	115.78	2.16
ef1-alpha (elongation factor 1 alpha)	Forward TCTGGTTGGCATGGAGACAA Reverse CAAGATGGCATCCAAAGCGT	129	60	0.99	-3.296	101.09	2.01
18S (18S rRNA)	Forward TACATGCAAACAGAGGTCCG Reverse CCATCGACAGTTGATAAGGC	191	60	0.993	-3.249	103.14	2.03
Vg.g1.i1 (vitellogenin, gene 1, isoform 1)	Forward CCATCCAGAACTGGATTCTC Reverse ATGAACGAGCTCGGTGTATG	197	60	0.999	-3.632	88.51	1.89
Vg.g1.i2 (vitellogenin, gene 1, isoform 2)	Forward CGTGAAGTCGCCTAGCTTTA Reverse TACCGAACGTATACAGCCTG	200	60	0.994	-3.372	97.95	1.98

Gene symbol and name	Primer sequence (5’ ➔ 3’)	Amplicon size (bp)	TM (°C)	R^2	Slope	Efficiency (%)	Amplification factor
Vg.g2.i1 (vitellogenin, gene 2, isoform 1)	Forward<br>AGCTCAGGTTACAAGCAAGC<br>Reverse<br>GGTGTTACTAGACCTCAACG	241	60	1	-3.594	89.78	1.90
VgR (vitellogenin receptor)	Forward<br>GGATACCGATGGACACGTTT<br>Reverse<br>TTCTGACTACGGTTGGTGTG	137	60	0.996	-3.388	97.31	1.97
Obp.g1.i1 (odorant-binding protein, gene 1, isoform 1)	Forward<br>CTACATAGGACTGGAATGCC<br>Reverse<br>ACGGATTTGCATTCGTCCAG	240	60	0.996	-3.229	104.03	2.04
Obp.g2.i1 (odorant-binding protein, gene 2, isoform 1)	Forward<br>ATGCTGAGGATCCATAGCGA<br>Reverse<br>AGTAGGTGTTTCCTGACGAC	113	60	0.996	-3.611	89.20	1.89
ObpR.g2.i1 (odorant-binding protein receptor, gene 2, isoform 1)	Forward<br>AAGAGAGCACCAACGTAGGT<br>Reverse<br>AAGTAGGAGTATCCCGCCTT	187	60	0.993	-3.266	102.39	2.02
ObpR.g3.i1 ((odorant-binding protein receptor, gene 3, isoform 1)	Forward<br>CTACGCTTCAGGCTATCAGT<br>Reverse<br>TTCTTCGGTTCGAGGTTGAG	134	60	1	-3.547	91.39	1.91
ACP (adult-specific cuticular protein ACP-20)	Forward<br>CTTCTAGCCATAGCGAATGC<br>Reverse<br>ACTCTGTGTTCTACCTGCTC	233	60	0.98	-3.566	90.73	1.91
Poly (polygalacturonase inhibiting proteins)	Forward	210	60	0.994	-3.218	104.53	2.05

**Gene symbol and name**	**Primer sequence (5' ➔ 3')**	**Amplicon size (bp)**	**TM (°C)**	**R^2**	**Slope**	**Efficiency (%)**	**Amplification factor**
	TATGGTGGAGGGGTGGTTTA<br>Reverse<br>GCTTTGACCAAACGCTCTGA						
Ega (egalitarian)	Forward<br>GTCAGGAACGTGATGGGTTT<br>Reverse<br>AAAGAGCCACCGAACTCAGA	213	60	0.997	-3.692	86.58	1.87
