# Supplementary material for: Transcriptional signature of host shift in the seed beetle Zabrotes subfasciatus
Source: Genet Mol Biol. 2024 Feb 5;47(1):e20230148. doi: 10.1590/1678-4685-GMB-2023-0148 (PMC10851049; doi:10.1590/1678-4685-GMB-2023-0148)
Supplement: Table S2 - [file 1415-4757-GMB-47-01-e20230148-s2.pdf]

Genetics and Molecular Biology
https://doi.org/10.1590/1678-4685-GMB-2023-0148

Supplementary Material to “Transcriptional signature of host shift in the seed beetle *Zabrotes subfasciatus*”

Table S2 - Descriptive statistics of two reference genes for qPCR assays of *Zabrotes subfasciatus* samples (calculated via BestKeeper.)

	rpl32	*ef1-alpha*
**N**	15	15
**geo Mean [CP]**	21.04	25.90
**ar Mean [CP]**	21.06	25.97
**min [CP]**	19.63	24.06
**max [CP]**	22.91	32.52
**std dev [± CP]**	**0.65**	1.30
**CV [% CP]**	**3.11**	5.00
**min [x-fold]**	-2.97	-3.62
**max [x-fold]**	4.20	101.74
**std dev [± x-fold]**	1.65	2.72
**coeff. of corr. [r]**	**0.933**	**0.982**
**p-value**	**0.001**	**0.001**

CP: crossing point; N: number of samples; geo Mean (CP): geometric mean of Ct; ar Mean (CP): arithmetic mean of Ct; min and max (CP): extreme values of Ct; std dev (± CP): standard deviation of the Ct; CV (% CP): coefficient of variance expressed as a percentage on the Ct level; min and max (x-fold): the extreme values of expression levels expressed as an absolute x-fold over or under-regulation coefficient; std dev (± x-fold): standard deviation of the absolute regulation coefficients; coeff. of corr. (r): coefficient of correlation.
